# The Feasibility, Appropriateness, Meaningfulness, and Effectiveness of Parenting and Family Support Programs Delivered in the Criminal Justice System: A Systematic Review

**DOI:** 10.1007/s10826-018-1034-3

**Published:** 2018-03-01

**Authors:** Victoria Troy, Kerri E. McPherson, Carol Emslie, Elizabeth Gilchrist

**Affiliations:** 1 Department of Psychology, Social Work and Allied Health Sciences, Glasgow Caledonian University, Cowcaddens Road, Glasgow, G4 0BA UK; 2 Glasgow Caledonian University, Glasgow, UK; 3 University of Worcester, Worcester, UK

**Keywords:** Parenting programs, Parenting, Implementation science, Implications for practice, Criminal justice system

## Abstract

Children whose parents are involved in the criminal justice system (CJS) are at increased risk of developing social, emotional, and behavioural difficulties and are more likely than their peers to become involved in the CJS themselves. Parenting behaviour and parent-child relationships have the potential to affect children’s outcomes with positive parenting practices having the potential to moderate some of the negative outcomes associated with parental involvement in the CJS. However, many parents in the CJS may lack appropriate role models to support the development of positive parenting beliefs and practices. Parenting programs offer an opportunity for parents to enhance their parenting knowledge and behaviours and improve relationships with children. Quantitative and qualitative evidence pertaining to the implementation and effectiveness of parenting programs delivered in the CJS was included. Five databases were searched and a total of 1145 articles were identified of which 29 met the review inclusion criteria. Overall, programs were found to significantly improve parenting attitudes; however, evidence of wider effects is limited. Additionally, the findings indicate that parenting programs can be meaningful for parents. Despite this, a number of challenges for implementation were found including the transient nature of the prison population and a lack of parent-child contact. Based on these findings, recommendations for the future development and delivery of programs are discussed.

Children whose parents have been imprisoned are often considered to be the ‘hidden victims’ of crime (Jardine 2017). While it is difficult to estimate the number of children affected (Miller 2006; Purvis 2013), the negative outcomes associated with parental imprisonment are well documented. For example, research has consistently found that children affected by parental imprisonment are at an increased risk of developing social, emotional, and behavioral difficulties (Dallaire 2007; Farrington et al. 2009). Moreover, they are more likely than their peers to have difficulties in school, to engage in delinquent behavior, and to become involved in the CJS themselves (Murray and Farrington 2005; Farrington et al. 2009; Trice and Brewster 2004). The complexity and extent of adversity makes it difficult to isolate the unique impact of parental imprisonment from other individual, family and community level factors. However, parental imprisonment can cause specific financial, social, and psychological burdens which may add to or exacerbate what is an already challenging upbringing (Arditti 2005). Growing up in such adverse conditions can have long term consequences which resonate into adolescence and adulthood and can further perpetuate the cycle of disadvantage and crime within families and communities (Murray and Farrington 2008).

While the impact of parental imprisonment on children is relatively well reported, the impact of imprisonment on parents has received considerably less attention. Parents in the CJS are more likely than non-offending parents to have experienced a multitude of difficulties including mental health problems, substance abuse, a history of difficult interpersonal relationships, and to have been recipients of harsh parenting (Buston et al. 2012). Qualitative research with parents in prison has shown that mental health difficulties are often worsened by separation from children and other family members (Brown and Bloom 2009) and because parents in prison often struggle to maintain contact with their children they have limited opportunities to play an active role in their children’s lives (Mignon and Ransford 2012). Many parents in prison will attempt to regain contact and/or custody of their children upon release (Brooks-Gordon and Bainham 2004) and the support that parents and families receive during the period of transition can have major implications for the readjustment process (Brown and Bloom 2009).

Parenting programs have been proposed as having the potential to mitigate the effect of parental imprisonment on the lives of families. Parenting programs are interventions that aim to improve outcomes for parents and children by enhancing parenting skills (Kaminski et al. 2008) either by working exclusively with parents or working with parents alongside other family members. They can be universal in delivery or target at risk families or families with specific issues (Kaminski et al. 2008). In recent years, a growing body of evidence has demonstrated the effectiveness of parenting programs in changing parenting attitudes and behaviors, improving parental mental health, and preventing or improving children’s social, emotional, and behavioral difficulties (Barlow et al. 2010; Dretzke et al. 2009; Furlong et al. 2013; Mejia et al. 2012). For parents in prison, parenting programs have the added potential of helping parents to build and/or maintain strong relationships whilst being separated from their children and families. This is important as enhancing family ties has been linked with better prisoner reintegration, reduced risk of recidivism, and better outcomes for children and families (Berg and Huebner 2011; Mills and Codd 2008; Sapouna et al. 2011). Consequently, investing in programs for parents in prison could have multiple advantages not only for the children of prisoners but for offenders themselves and society more broadly (Poehlmann et al. 2008).

Given their potential benefits, there is increasing interest in the ways in which parenting programs can be designed and delivered effectively and efficiently within a prison environment. To date, much of the research has focused on the potential effectiveness of parenting programs and although there is evidence about the barriers and facilitators to implementing parenting programs in the general population (McPherson et al. 2017; Whittaker and Cowley 2012) far less is known about the mechanisms driving successful implementation of parenting programs within the context of prisons. In regard to parenting programs for parents in the CJS, the evidence base is hampered: firstly, by a lack of consensus about what constitutes a parenting program (Sandifer 2008); and secondly, by a paucity of empirical evidence relating to how programs are implemented, evaluated, and experienced by key stakeholders, including the parents undertaking them (Eddy et al. 2008). As such, the current body of evidence is weak and this limits our understanding of the type of intervention most likely to be effective and under what conditions implementation is likely to be successful. The inadequacies of the evidence base extend beyond programs delivered in prisons but also to those delivered at other stages of the CJS (i.e., upon release).

Thus, the aim of the current systematic review was to inform an evidence based approach to the delivery of parenting programs in the CJS by identifying and synthesizing evidence regarding the feasibility, appropriateness, meaningfulness, and effectiveness (FAME) of existing programs.

## Method

Given that the purpose of this review was to synthesize evidence about feasibility, appropriateness, meaningfulness, and effectiveness a mixed study review was adopted. This enabled the inclusion of qualitative, quantitative, and mixed method studies for analysis. For the purpose of this review: *feasibility* (F) refers to whether it is possible to deliver a parenting program within a criminal justice context and includes the physical and cultural practicality of implementation; *appropriateness* (A) refers to the extent to which the program is relevant for parents in the CJS; *meaningfulness* (M) refers to the perception of the program as positive and useful, or otherwise, by recipients; and, *effectiveness* (E) refers to the extent to which the parenting program achieved its intended effect. The review and definitions of feasibility, appropriateness, meaningfulness, and effectiveness were informed by the Johanna Briggs Institute (JBI) model of evidence-based healthcare (Pearson et al. 2005).

### Criteria for Inclusion

#### Eligible designs

Qualitative studies, quantitative studies with and without a control group, and mixed methods studies were included in this review. Published and peer reviewed studies were eligible for inclusion. Non-empirical studies, case studies and economic evaluation studies were excluded.

#### Types of participants

The target population was parents who had received a parenting program as part of their involvement in the CJS. Included in this were parents who volunteered to participate in parenting programs whilst in prison or in the period described by the study authors as ‘following release from prison’, and those who were mandated to take part by the courts. Studies that focused on parents of children and adolescents (3–18 years) were included. Programs targeting parents of children and infants less than 3 years old were excluded because the needs of these parents, and support available to them, may be different; for example, in some countries mothers may be able to keep their infant with them in prison. Consequently, the nature and content of programs for parents of infants may differ from those targeting parents of older children.

#### Types of programs

Any parenting program (see definition provided on p.3) where the parent in the CJS was the main recipient was included. Therefore, programs were excluded if the primary recipient was children of parents in the CJS or kinship carers. Programs were also excluded if they were delivered to parents because of their child’s offending behavior. Programs were not excluded on the basis of delivery format or theoretical framework.

#### Types of outcomes

Evidence pertaining to the FAME of parenting programs was sought for this review. To assess feasibility, appropriateness and meaningfulness, data about implementation (e.g., recruitment, retention, and program adaptations) as well as recipient and facilitator perceptions of the program were extracted. To assess effectiveness, outcome data which was measured using standardized instruments relating to parenting, parent psycho-social health, children’s social emotional and behavioral development, family cohesion and stability, and parental risk factors (e.g., substance use, recidivism) were extracted.

### Search Strategy

#### Electronic searches

Five databases were searched for this review and searches were completed in January 2016. Three were searched via the ProQuest platform: PsychInfo, Medline, and the National Criminal Justice Reference Service (NCJRS). The Cumulative Index to Nursing and Allied Health Literature (CINAHL) was searched via EBSCO and Science Direct was searched directly. The search strategy was tailored for each search engine but contained a combination of MESH/subject headings and keywords. The search included: parent (e.g., parent, mother, father, caregiver); intervention (e.g., parent training, parent program, parent intervention, family program, family therapy, family intervention); and, context (e.g., prison, criminal justice, parole, probation, correctional facility, imprisoned) search terms. Full search strategies are available on request from the authors. Hand searches of work known to the authors and the reference lists of relevant systematic and non-systematic reviews were also undertaken. The search was limited to literature published from 1970 onwards and had to be published in English.

### Data Collection and Analysis

#### Selection of studies

Following the searches, all references were managed in *RefWorks*^©^ and duplicates were removed. The titles and abstracts were reviewed and studies that did not fit the inclusion criteria were rejected. To ensure the screening process was rigorous screening was conducted by two independent reviewers. Following this, studies that met the inclusion criteria were retrieved in full. Paired reviewers conducted screening of the full text articles; the principal reviewer (VT) screened all articles and the other three members of the research team (KM, CE, & LG) received a sub-set. In cases where there was a disagreement regarding suitability, the two reviewers discussed the article until consensus was reached.

#### Data extraction

Data extraction was conducted using a review-specific extraction form (available on request from the lead author). Data extracted included the: aims and purpose of the study; population characteristics (i.e., relationship status, education level, history of abuse, mental health, and/or substance abuse, offending information, information about children,and family structure); program characteristics (i.e., theoretical underpinnings, content, delivery); and, study characteristics (i.e., methods, analysis, and findings). VT undertook all data extraction with each one being quality checked by KM, CE, or LG.

#### Quality appraisal of included studies

Quality appraisal was undertaken using a review-specific tool (available on request from the lead author). The checklist was informed by previous systematic reviews that adopted a mixed study approach (Bunn et al. 2008; Glover et al. 2015). The quality assessment was based on 20 items relating to: study design, selection bias, data collection, data analysis, and reporting of outcomes. Each article was awarded a quality rating of *high*, *moderate*, or *low* depending on the percentage of answers coded as meeting the criteria (scores ≤ 35% were coded ‘low’; 35–69% were coded ‘moderate’; and, ≥70% were coded ‘high’). The principal reviewer (VT) quality assessed all articles and the other three members of the research team (KM, CE, & LG) checked for accuracy within their sub-set. Any disagreements were resolved through discussion and/or through consultation with a third reviewer. Quality appraisal was undertaken to aid interpretation of findings and assist in determining the strength of the conclusions drawn; no study was excluded based on the results of the quality assessment.

#### Analysis and synthesis

The mixed-method approach of the review and the heterogeneity of outcomes precluded the use of meta-analysis. After extraction, data were grouped according to the element of FAME it related to and summarized and synthesized independently. In what follows, the results relating to feasibility, appropriateness, meaningfulness and effectiveness are presented separately.

## Results

**Fig. 1:**
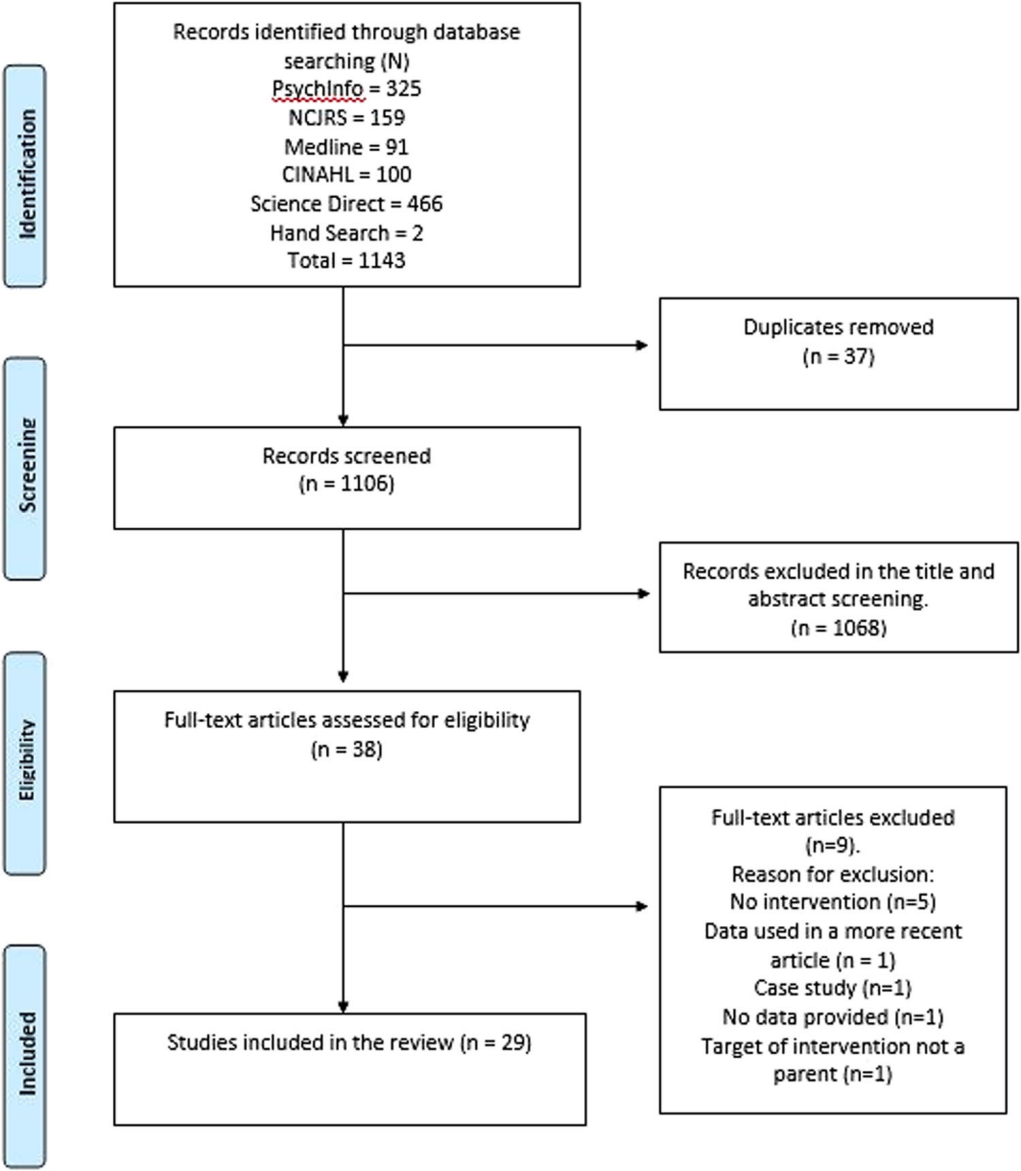
: PRISMA flow diagram of study selection

As shown in Fig. 1, initial database searches identified 1143 papers and a further two papers were identified through hand searches. Following the removal of duplicates and the screening of titles and abstracts, full text screening was undertaken on 38 articles. A further nine articles were excluded at this point, the reasons for which are outlined in Fig. 1. In total, 29 studies were identified as eligible for inclusion in this review.

### Description of Included Studies

To aid reporting, all studies were allocated a study reference number and this is used in what follows. The reference numbers and characteristics of the included studies are contained in Table 1 which is ordered according to the studies quality rating (highest to lowest quality).

**Table 1:** Study characteristics

ID number Available FAME data	Quality Rating	Author Country, setting	Study design Data collection points Target population (participants recruited (R), included in analysis (A), attrition (%))	Parenting program
1 AME	High	Eddy et al. (2013) USA, Prison	RCT Pre and post intervention Mothers and fathers (*R* = 359, A = 255, 29%)	Parenting Inside Out (PIO)
2 E	High	Loper and Tuerk (2011) USA, Prison	RCT Pre and post intervention Mothers (*R* = 176, *A* = 90, 49%)	Parenting From Inside: Making The Mother–Child Connection
3 AE	High	Menting et al. (2014) the Netherlands, Prison and post-release	Non-equivalent control group design Pre intervention, post intervention, home visit, and follow up Mothers (*R* = 113, *A *= 91, 19%)	Incredible Years
4 FAME	High	Miller et al. (2014)USA, Prison	Mixed-methods, cohort study Pre and post intervention Mothers (*R* = 45, *A* = 22, 42%)	Parenting While Incarcerated
5 AE	High	Shortt et al. (2014) USA, Prison and post-release	Matched control group design Pre and post intervention, follow up (*R* = 47, *A* = 47 post-test; 38 follow up, 19%)	Parenting Inside Out and Emotions: Taking Care of Yourself and Your Child When you go Home
6 FME	High	Skar et al. (2014)Norway, Prison	Mixed-methods, non-equivalent control group design Pre and post intervention Fathers (*R* = 129, *A* = 61, 53%)	International Child Development Program
7 FAME	Moderate	Block et al. (2014) USA, Prison	Mixed-methods, non-equivalent control group design Pre and post intervention Fathers (*R* = 309^a^, *A* = 413, 17%)	Inside Out Dad
8 E	Moderate	Frye and Dawe (2008) Australia, Community	Single-group design Pre and post intervention, follow up Mothers (*R* = 12, *A* = 8, 33%)	Parenting Under Pressure (PUP)
9 FE	Moderate	Harris and Landreth (1997) USA, Prison	Non-equivalent control group design Pre and post intervention Mothers (*R* = 51, *A* = 22, 57%)	Filial Therapy
10 FE	Moderate	Kennon et al. (2009) USA, Prison	Mixed-methods, single-group design Pre and post intervention, follow up Mothers (*R* = 66, *A* = 57, 14%)	No formal title
11 FE	Moderate	Landreth and Lobaugh (1998) USA, Prison	Matched control group design Pre and post intervention Fathers (*R* = 32, *A *= not specified, unable to calculate attrition)	Filial Therapy
12 E	Moderate	Robbers (2005) USA, Prison	Non-equivalent control group design Pre and post intervention Fathers (*R* = 122, *A* = 87, 29%)	FairFax Fatherhood Program
13 FE	Moderate	Sandifer (2008) USA, Prison	Non-equivalent control group design Pre and post intervention Mothers (*R* = 161, *A* = 91, 43%)	Rebonding and Rebuilding: A Parenting Curriculum
14 FME	Moderate	Scudder et al. (2014) USA, Prison	RCT (I: PCIT, C: Existing Program) Pre and post intervention Mothers (*R* = 82, *A *= 69, 16%)	Parent–Child Interaction Therapy
15 E	Moderate	Surratt (2003) USA, Substance abuse treatment facility	Matched control group design Pre and post intervention Mothers (*R* = 59, *A* = 46, 22%)	Key Village Parenting Class
16 E	Moderate	Wilczak and Markstrom (1999) USA, Prison	Non-equivalent control group design Pre and post intervention Mothers (*R* = not specified, *A* = 42, unable to calculate attrition)	Systemic Training for Effective Parenting
17 E	Moderate	Wilson et al. (2010)USA, Prison	Single-group design Pre and post intervention Mothers and Fathers (*R* = 184, *A* = 150, 18%)	Parenting From Prison
18 E	Low	Bushfield (2004) USA, Custodial bootcamp	Mixed-methods, single group design Pre and post intervention Fathers (*R* = not specified, *A* = 32, unable to calculate attrition)	North Idaho Corrections Institute
19 AE	Low	Cornille et al. (2005) USA, Custodial bootcamp	Mixed-methods, single group design Pre and post intervention Fathers (*R* = not specified, *A* = 63, unable to calculate attrition)	Dads Actively Developing Stable Families Family Project
20 E	Low	Gonzalez et al. (2007) USA, Prison	Single-group design Pre and post intervention Mothers (*R* = 219, *A* = 191, 13%)	Parenting in Prison
21 E	Low	Harrison (1997) USA, Prison	Matched control group design Pre and post intervention Fathers (*R* = not specified, *A* = 30, unable to calculate attrition)	No formal title
22 AME	Low	LaRosa and Rank (2001) USA, Prison	Mixed-methods, single-group design Pre and post intervention Fathers (*R* = 26, *A* = 23, 12%)	Real life Parenting Skills Program
23 FAME	Low	McCrudden et al. (2014) UK, Prison	Mixed-methods, single-group design Pre and post intervention Fathers (*R* = 24, *A* = 18, 25%)	Being a Dad Program
24 M	Low	Meek (2007) UK, Prison	Qualitative, retrospective evaluation study Post intervention Fathers (*R* = not specified, *A* = 75, unable to calculate attrition)	No formal title
25 FE	Low	Moore and Clement (1998) USA, Prison	Mixed-methods, non-equivalent control group design Pre and post intervention Mothers (*R* = not specified, *A* = 40, unable to calculate attrition)	Mothers Inside Loving Kids
26 E	Low	Palusci et al. (2008) USA, Multiple	Non-equivalent control group design Pre and post intervention Mothers and Fathers (*R* = unclear, *A* = unclear, unable to calculate attrition)	Helping Your Child Succeed
27 FM	Low	Rossiter et al. (2015) USA, Prison	Mixed-methods, retrospective evaluation study Post intervention Mothers (*R* = not specified, *A* = 32, unable to calculate)	Mothering at a Distance Program
28 E	Low	Thompson and Harm (2000) USA, Prison	Single-group design Pre and post intervention Mothers (*R* = not specified, *A* = 104, unable to calculate attrition)	Parenting from Prison Program
29 FME	Low	Urban and Burton (2015) USA, Prison	Non-equivalent control group design Pre and post intervention Mothers (*R* = unclear, *A* = unclear, unable to calculate attrition)	Parents and Their Children: Turning Points

^a^ Number of participants recruited to control group unknown

#### Methodological quality of included studies

Six studies were rated as high quality (1–6), 11 as moderate quality (7–17), and 12 as low quality (18–29). The reporting of findings was particularly poor in one article (28), as it contained numerous inconsistencies between values displayed in tables and those quoted in the narrative; multiple post-hoc sub-group analyses which diluted the integrity of the findings; and, frequent references to data and findings reported in an earlier paper. Consequently, some data from this study have been omitted from this review (see below for detail).

#### Study designs

The majority of studies included in this review were quantitative (*N* = 18) (see Table 1). Ten studies were mixed methods (4, 6, 7, 10, 18, 19, 22, 23, 25, 27) and one study was entirely qualitative (24). The majority of the studies (*N* = 14) with a quantitative element had adopted a quasi-experimental, between group design (3, 5–7, 9, 11–13, 15, 16, 21, 25, 26 29). Nine were single group designs reporting pre-and-post program data (8, 10, 17–20, 22, 23, 28), three were randomized controlled trials (1, 2, 14), and two were retrospective studies reporting post-program information only (24, 27). The final study was a cohort study (4). Follow-up data was collected in four studies (3, 5, 8, 10)

#### Geographical spread and setting

The studies were conducted primarily in the USA (1, 2, 4, 5, 7, 9–22, 25, 26, 28, 29) with the remainder undertaken in the UK (23, 24), Australia (8, 27), the Netherlands (6) and Norway (3). All but one study reported on programs delivered in custodial settings (*N* = 28). The remaining study reported on a program delivered in the community to parents who were on community custody order or who had recently been released from prison (8).

#### Sample

Just over half of the studies (*N* = 16) included mothers only (2–5, 8–10, 13–16, 20, 25, 27–29), 10 included fathers only (6, 7, 11, 12, 18, 19, 21–24), and three included a combination of both mothers and fathers (1, 17, 26). Sample sizes varied considerably across the included studies and it was not always possible to determine whether the numbers reported referred to those initially recruited or those in the final sample; available data is reported in Table 1. The reported number of recruited participants ranged from 12 (8) to 476 (7). The majority of studies (*N* = 22) conducted their analysis on relatively small samples with fewer than 100 participants (2–6, 8–10, 12–16, 18, 19, 21–25, 27).

Reporting of participants’ sociodemographic details, such as age, ethnicity, and education level, tended to be poor. Information was either not provided or reported in a way which made interpretation difficult. The reporting about participants’ children was also poor. For example, less than half of the studies (*N* = 13) included any information about the age of children (1–3, 5, 6, 9–11, 13, 21, 23, 25, 28). Where information about the average age of children was provided, the figures ranged from 5 (9) to 10.5 years (2). Only four studies mentioned the care status of children (9, 14, 17, 28), the majority of children in these studies were in kinship care; however, it was unclear whether this was court mandated or voluntary.

#### Program content and delivery

Three programs were evaluated in more than one study: Parenting Inside Out (1, 5); Filial Therapy (9, 11); and Parenting from Prison (20, 17).

##### Aims, intensity, and content of programs

All studies reported on the delivery of programs exclusively to parents involved in the CJS; however, some of these programs have previously been delivered to wider populations (e.g., Incredible Years). Twelve studies failed to clearly report the aims of the program under investigation. In studies where the aims were reported, these often referred to the program as affecting multiple outcomes. The most commonly reported aim was to improve parent-child interactions and/or parent-child relationships by improving general parenting knowledge and skills, and by increasing parents ability to understand and respond to children’s needs (6, 7, 9, 11 - 14, 16, 25- 27). Less frequently acknowledged aims included: improving parental wellbeing (2, 25); preventing or alleviating children’s social, emotional, and behavioural difficulties (1, 6); improving relationships between caregivers (2, 12); providing problem solving skills for use upon release (5); and, reducing the risk of child maltreatment and promoting parental recidivism (25).

##### Program intensity

Nine studies failed to provide information regarding the average length of sessions; therefore, for these studies, the intensity of program refers to the number of sessions delivered and this ranged from 10 to 20 sessions. Where there was information about the number of hours included, the programs ranged in intensity from approximately 7.5 h (22) to 120 h of contact (5). Two programs also offered parents support following release from prison (3, 5). In one, this post-release support was a core component of the program and included 6 h of home visits (3); in the other, post-release support was optional and available for up to 6 months (5). A case management approach was adopted in one study (8). The program offered in this study included 10 core modules; however, the intensity of the program varied in relation to parent and family needs with the addition of adjunctive support available to families with more complex needs.

##### Topics covered by programs

The most frequently reported topics were disciplining children (*N* = 23), communication (*N* = 20) and general positive parenting concepts (*N* = 15). Disciplining children and positive parenting topics had very little detail regarding what information was provided or what skills were taught. Greater detail was available regarding the knowledge and skills covered as part of the communication topic. In addition to general communication skills, specific skills included: how to communicate about emotions; how to communicate from afar; how to talk about an offence with children; and, how to communicate with other caregivers.

Other common topics included child development (*N* = 14), emotion regulation skills (*N* = 9), and topics specifically related to being a father (*N* = 5). None of these topics were particularly well described therefore the content of the sessions are difficult to determine.

Some programs contained information about topics that are known to influence parenting but that are not directly related to parenting knowledge, attitudes, or behaviors; for example, nutrition, finance, health and safety, relationship advice, and information about parental substance abuse.

### Feasibility, Appropriateness, and Meaningfulness

None of the included studies assessed feasibility or appropriateness using quantitative methods. Previous research has used attrition rates as a proxy measure of feasibility; however, this was not possible with the data available for this review because variations in reporting limited our ability to calculate or meaningfully interpret attrition rates (available data is presented in Table 1). Therefore, in this review, the findings relating to feasibility and appropriateness rely upon qualitative evidence only.

#### Feasibility

Only one study (4) explicitly reported assessing feasibility. However, relevant information was extrapolated from a further 11 studies where issues relating to the recruitment, retention, and evaluation of programs were discussed (6, 7, 9–11, 13, 14, 23, 25, 27, 29). Three key areas relating to feasibility were identified and are discussed below: the transient nature of the prison population; the nature and environment of delivery; and, parent-child separation.

The transient nature of the prison population was described as particularly problematic in four studies (4, 7, 13, 27). The inability to accurately predict prisoners’ release dates was described as a major challenge for recruitment and retention by facilitators in one study (7). In addition, the instability of the prison population (i.e., frequent turnover and transfer of prisoners) was considered problematic for the implementation and evaluation of programs (4, 13, 27) and led to recipients reporting that they felt ‘letdown’ by constant changes to groups (4). Delivering shorter and more intense iterations of the program (27) or the delivery of topics on a stand-alone basis (4, 13) were offered as potential solutions to mitigate the difficulties associate with the transient prison population.

Reports by program facilitators in two studies (4, 7) indicated that the environment in which programs were delivered is particularly important. In both, the facilitators acknowledged that there was a need to create safe environments conducive to in-depth discussion. However, large group sizes (7) and participant apprehensions (4) were described as potential barriers that have the ability to undermine positive group processes and negatively impact on group dynamics.

The most frequently reported barrier to feasibility was the lack of parent-child contact and the impact this had for skill building opportunities. Infrequent or non-existent contact presents challenges to the successful delivery of parenting programs as the opportunity to rehearse and build skills becomes limited. To reduce the likelihood of infrequent or non-existent contact, eight studies included enhanced visitation for children as part of the program package (4, 6, 9, 11, 13, 23, 25, 29). What constituted enhanced visitation varied between studies but was likely to include more frequent visits, longer visits, and visits that permitted physical contact. Enhanced visitation meant that parents could rehearse their newly learnt skills and receive feedback from the facilitators and this was portrayed positively within the studies. No study explicitly referred to the impact of enhanced visitation on the outside carer. However, in one study (4), the importance of having a community partner agency was described as vital for overcoming potential barriers associated with the implementation of enhanced visitation.

#### Appropriateness

As with feasibility, only one study (4) explicitly investigated the appropriateness of parenting programs. However, relevant information was extrapolated from a further five studies where participants, facilitators, and/or authors commented on the appropriateness of materials and program topics, or where their recommendations for program improvements were discussed (3, 7, 19, 22, 23).

Two studies referred to the appropriateness of parenting programs from the perspective of facilitators (4, 7). In both studies, facilitators reported confidence in the program and accompanying materials; however, the authors did not provide detail as to how this confidence was developed and maintained.

Four studies highlighted the importance of ensuring that information and examples met the needs of recipients (4, 7, 22, 23). In particular, it was recognized that parenting information should cover a wide range of developmental stages, cover a wide range of topics, provide opportunities to increase family involvement and engage in practical activities such as role play, homework clubs, and video messages.

Seven studies made reference to the use of printed program materials (i.e., manuals and handouts) for program recipients (3–5, 7, 9, 19, 23). Of these, only two (7, 19) assessed their appropriateness. The materials were viewed positively by participants in one study (7) whereas they were described as ‘too academic’ in the other (19); there was not enough information regarding the content of the materials to interrogate the contrasting views. However, participants who viewed it as too academic suggested the inclusion of digital workbooks with practical exercises as a way to improve the program as a whole so this may provide insight regarding preferences for the formatting of program material (19). The need for materials to be easily comprehensible was noted in two studies (3, 4) but it was unclear whether this was the opinion of the facilitators, participants, or authors. Taken together, this suggests that the format of printed program materials may be an important consideration.

#### Meaningfulness

Eleven studies reported on parents’ satisfaction with the program (1, 4, 5, 6, 7, 14, 22–24, 27, 29). Overall, there was a strong consensus across the studies that parenting programs could be meaningful for parents in prison. Evidence from five studies using satisfaction scales, indicated high levels of satisfaction (1, 4, 5, 14, 27). Moreover, qualitative findings suggested that participants were happy with the information they received, that the knowledge and skills were useful, and that their confidence in their parenting had improved (1, 4, 6, 7, 22, 24, 27, 29). Topics such as setting realistic expectations, coping with addiction, and managing complex emotions were regarded as particularly useful by participants in one study (4). Receiving support in managing emotions was also rated favorably by participants in another study (5) who received an emotions-based program in addition to a parenting program. Sessions on discipline, empathy, child development, and positive parenting practices were the most appreciated topics according to participants in two studies (22, 24). In addition to specific topics, participants regarded opportunities to engage in practical activities as especially meaningful (23).

The quality of the facilitator was also regarded as important to participants. In particular, the (perceived) level of knowledge about the topic, and an understanding of the unique needs of parents in prison were considered essential (22, 24, 27).

In seven studies, participants expressed a desire for either an increase in the number/length of sessions, or additional support after the program ended (4, 6, 7, 22–24, 27). Although no data was available to interrogate this fully, a desire for an extended program or further support could be interpreted as parents experiencing the programs in a positive way and wanting to continue with the parenting program. In contrast, it might be indicative of an unmet need, but one that parents perceive can be addressed with additional parenting input. Alternatively, it could reflect the prison context in which prisoner parents seek out opportunities to engage in activities of any type. Indeed, this may account for the discrepancies voiced by participants in one study (24) whom on one hand expressed a desire for further support but on the other stated they neither wanted nor needed support upon release.

#### Effectiveness

Information about the design of studies assessing effectiveness, including information about comparison groups, is available in Table 1. All except two studies (24, 27) explored the effectiveness of programs. Twenty studies reported assessing changes to parenting attitudes (*n* = 17), knowledge (*n* = 5) and/or behavior (*n* = 3) following participation in a parenting program. All 20 used self-report measures to assess attitudes and knowledge, but behavior was assessed both by parental self-report and through others’ reports (e.g., teachers and children). As noted above, difficulties related to reporting in one study (28) meant that the findings relating to parenting attitudes could not be included therefore changes to parenting attitudes is based on findings from 16 studies only.

##### Parenting attitudes

Twelve of 16 studies reported improvement in parenting attitudes post-program. Half of the studies utilized a between group design; three reported significant improvements for the treatment group but not the control (12, 13, 21); however, only one of these reported a significant group × interaction (21). Two reported improvements for both groups but significantly higher scores for the treatment group (9, 11). One study (14) reported significant improvements for parents in the treatment as usual group, but not for those who received Parent Child Interaction Therapy. A significant interaction effect was reported in one study (6) which indicated improved attitudes for the non-offending comparison group but worsened attitudes for the prison group. The remaining single-group studies reported improvements based on positive changes between pre-and-post program test scores (4, 17–19, 22, 26).

When assessing attitudes, four studies reported findings based on a calculated total score only (9, 12, 17, 21), the remaining seven reported findings for total scores and attitude type sub-scales (4, 11, 13, 14, 19, 22, 26). Where different attitude types were assessed, studies reported a significant improvement in discipline-related attitudes (e.g., participants less likely to report a belief in corporal punishment, less likely to report inconsistent discipline, and were more likely to avoid harsh and/or physical punishment after participating in the parenting program). Four studies also reported improved understanding of child development and attitudes towards age appropriate expectations (13, 14, 22, 26) and two reported an improved ability to recognize and respond to children’s needs (13, 26). Only one of these studies (14) reported significant group × time effects. Three studies did not find any significant changes in parenting attitudes (7, 15, 25) but the authors in one (15) suggested this was indicative of a ceiling effect given that pre-program scores reflected normative responses. Results from four studies (17, 19, 22, 26) indicated a need/response paradigm as gains were greatest for those who reported fewer positive attitudes pre-program. Overall, the findings suggest that parenting programs can be effective in changing parenting attitudes for parents in the CJS.

##### Knowledge

Five studies assessed changes in knowledge either immediately following each program session (29) or after program completion (7, 16, 17, 25). All studies used a program specific tool to measure knowledge and all reported significant improvements; however, only one study reported a significant group × time effect (7).

##### Parenting behavior

Four studies assessed changes to parenting behavior. Two assessed changes to parents’ behavior through the observation and coding of parent-child play by researchers blind to condition (9, 14) and two assessed changes by way of parental self-report (3, 10). All four studies reported improvements immediately following participation in the parenting program but only two (3, 14) reported significant group × time effects. Observational findings indicated improvements relating to attention, communication, and child-centered play (9, 14). Self-reported findings indicated a reduction in the use of inconsistent discipline (3), a reduction in hostility towards children and increased affection and warmth (10). Both studies using self-report measures (3, 10) included a follow up data collection point and both reported that improvements were maintained.

##### Communication and contact

The type and frequency of contact between parents and children was assessed in five studies (2, 7, 10, 12, 17). Of these, four reported an increase in the frequency of contact following the parenting program (2, 7, 12, 17); however, only one reported a significant group × time effect (7). In addition to increased frequency between parents and their children, participants in one study (2) reported more frequent communication and consultation with their children’s outside caregiver. In addition to frequency of communication, one study (1) assessed parents’ perceptions about whether the contact had a positive, negative, or neutral influence on their children. Parents who received the program viewed their interactions more favorably than parents in the control group. These findings expand on those relating to frequency as they highlight the potential for wider benefits in relation to contact and communication.

##### Child functioning

Children’s behavior was assessed in four studies by way of parental report (3, 8, 9, 11). Parents in all four studies reported improvements in their children’s behavior following the program. However, only one of these studies reported a significant group × time effect (3) and only for intensity of disruptive behaviors not frequency of these behaviors. One of the studies (3) also included reports from teachers who were blind to condition; no significant difference between the parenting program and control group was found.

##### Indicators of child health and wellbeing

The impact of parenting programs on children’s health and wellbeing was assessed in two studies (11, 21). Children’s perception of themselves was assessed via self-reported measures in both. In one (11), the children whose parents had participated in the parenting program reported significantly more positive views about themselves post-program; no significant differences were found in the other (21).

##### Indicators of parent health and wellbeing

In total, 13 studies assessed changes to parents’ health and wellbeing using a variety of different indicators, including: depression (*N* = 3), general health and wellbeing (*N* = 4), self-esteem (*N* = 5) and stress (*N* = 6).

Two of the three studies that assessed depression reported significant reductions in levels of depression after participating in the parenting program (1, 5). However, only one of these (1) reported a significant group × time effect. Both studies evaluated the effectiveness of the Parenting Inside Out program, which has a high level of program intensity and could indicate the need for intensive programming. The differential impact of the program on mothers and fathers depression was assessed in one (1); findings show that despite improvements, mothers continued to have significantly higher levels of depressive symptoms compared to fathers at post-program.

Three of the four studies that assessed parents’ general health and wellbeing reported significant improvements following participation (2, 5, 8) and two of these (2, 5) reported significant group × time effects. The fourth study (6) reported significant deteriorations in quality of life and life satisfaction following participation in the parenting program.

Three of the five studies that assessed self-esteem reported significant improvements following participation (10, 17, 28). One study (28) investigated the mediating effects of parent-child contact and found self-esteem only improved significantly for mothers who had frequent contact with their children. In the only study to undertake a follow up, improvements were maintained 2 months after program completion (10).

Five of the six studies that reported on stress found significant reductions in stress levels following program participation (1, 2, 8, 11, 14); however, only one of these studies reported a significant group × time effect (1). In the only study to undertake a follow up, improvements were maintained 3 months after program completion (8).

Five studies explored the impact of parenting programs on parental confidence and self-efficacy (6, 7, 17, 20, 23). All five found that parents reported greater confidence in their parenting abilities post-program; however, the increase was not significant in one study (6). Moreover, only one of these studies reported a significant group × time effect (7). One study reported significant improvements to parental satisfaction, which included satisfaction with children’s behavior, parenting ability, and the parent-child relationship (17).

### Additional Support

Two studies (3, 5) reported on programs with additional support over and above the parenting program, one (3) reported that the inclusion of individual home visits added to the positive effects of the group program and produced continued decreases in the use of inconsistent discipline. The other (5) reported that support following release led to additional and significant reductions to emotional dysregulation and recidivism but additional support did not appear to be associated with greater health and wellbeing improvements more generally. Overall, additional support was shown to enhance the effects of the parenting programs.

## Discussion

Twenty-eight of the 29 included studies reported on programs delivered within a prison context and overall the review findings provide useful insights to support the development, evaluation and implementation of parenting support programs for incarcerated parents. In addition, this review has highlighted where gaps in knowledge exist, most notably in the paucity of evidence about parenting programs for parents in the wider CJS.

### Effectiveness

The majority of studies included in the review focused exclusively on investigating the effectiveness of parenting programs. Overall, the evidence suggests that parenting programs can be effective in promoting positive outcomes especially in relation to parenting attitudes, parenting knowledge, and indicators of parental health and wellbeing. Specifically, findings indicate that parenting programs can be effective for improving attitudes regarding discipline (Cornille et al. 2005; Landreth and Lobaugh 1998; LaRosa and Rank 2001; Miller et al. 2014; Palusci et al. 2008; Sandifer 2008; Scudder et al. 2014). In the context of parental health and wellbeing, programs were found to be particularly beneficial for depression (Eddy et al. 2013; Shortt et al. 2014), self-esteem (Kennon et al. 2009; Thompson and Harm 2000; Wilson et al. 2010), and stress (Eddy et al. 2013, Frye and Dawe 2008; Landreth and Lobaugh 1998; Loper and Tuerk 2011; Scudder et al. 2014). Parenting programs were also shown to increase the frequency of communication between parents and children (Block et al. 2014; Loper and Tuerk 2011; Robbers 2005; Wilson et al. 2010) as well as between parents and other outside caregivers (Loper and Tuerk 2011). Despite these positive findings, the evidence regarding the effectiveness of programs on parenting behavior, children’s behavior and on children’s health and wellbeing was limited.

Importantly, even though there was a range of positive benefits across parent outcomes, reports of unintended consequences were also noted. Most strikingly, Skar et al. (2014) found that men’s attitudes to parenting became more negative and their mental health and wellbeing worsened following participation in the parenting program. Unfortunately, no evidence was available to investigate why this might be the case; however, the authors of the study hypothesized that the unintended pattern of outcomes could be explained by participating fathers engaging in more realistic appraisals of their parenting which may have led to feelings of guilt and remorse. Indeed, social comparison theory would suggest that when offered models of ‘good parenting’, modeled through parenting programs, individuals who perceive that they fall short of these may experience negative internalizing processes (Festinger 1962). Acknowledging the potential for unintended consequences is an important yet often neglected component of evaluations (Bonell et al. 2014). Future research into the development and implementation of parenting programs for parents in the CJS should be sensitive to unintended consequences, the implications of these for parents and families and, where appropriate, employ strategies to mitigate or alleviate these consequences.

### Feasibility, Appropriateness and Meaningfulness

The reporting of evidence describing the feasibility, appropriateness, and meaningfulness of programs was sparse. However, this is perhaps unsurprising given that until very recently the focus of intervention research was on establishing effectiveness rather than giving careful consideration to factors that might impact on the optimization of implementation. That said, sensitive to emerging implementation science knowledge base, this review was able to report on some of the unique factors associated with the delivery and evaluation of programs in prison and provide important lessons for future practice.

First, the studies included in this review described the transient nature of the prison population as a primary concern and highlighted that traditional methods for delivering parenting programs (i.e., the tendency for delivery to be in a sequential manner, where each element builds upon previously delivered content) may not be practical, nor best practice in the prison context. Within the studies in this review, Miller et al. (2013) and Sandifer (2008) suggest adapting programs so that sessions can be delivered on a stand-alone basis rather than requiring continuity in attendance may mitigate some of the difficulties. Adopting a flexible delivery approach would allow for continuous recruitment to the program, thereby maximizing sustainability; it would enable parents to participate irrespective of the length of their sentence; and, it would also allow parents who miss sessions due to scheduling conflicts (e.g., court attendance) to continue with their engagement. However, it should be noted, that while stand-alone sessions may increase engagement and retention of participants there may be undesired consequences. For example, maximum flexibility might encourage parents to opt out of sessions which will dilute the intensity of the program and may cause group instability (Whittaker and Cowley 2012). In addition, while flexibility may be beneficial for successful implementation, allowing maximum flexibility is problematic in the context of evaluation research as it limits opportunity to generate credible evidence about the effectiveness of programs, and understand more nuanced issues such as minimal sufficiency and the cost-effectiveness of programs. To avoid or limit potential concerns, implementing agencies and facilitators may wish to utilize strategies to support learning and prolong engagement such as setting a mandatory minimum, providing comprehensive resources, encouraging peer support, and providing appropriate incentives.

Second, the studies included in this review highlighted the important role played by facilitators as they were found to impact the way recipients responded to the program (LaRosa and Rank 2001; Meek 2007; Rossiter et al. 2015) and were considered essential in the management of group dynamics (Miller et al. 2013). Previous research has described the strength of the relationship between facilitators and recipients as having a profound impact on initial and prolonged engagement in parenting programs (Fixsen et al. 2005; McPherson et al 2017) as well as being associated with better treatment outcomes (Marsh et al. 2010). Ensuring a good relationship between facilitators and recipients is a vital component in the successful delivery of programs (Ingoldsby 2010) and may be particularly important in a prison context where distrust of professionals, unequal power dynamics, and a reluctance to engage in programs are common problems (Morgan et al. 2007). In addition to relational qualities, studies in this review emphasized the need for facilitators to accurately recognize and respond to parents’ concerns and for program recipients to perceive that facilitators understand their needs. These findings mirror what has been discussed within the family support literature on working with other marginalized groups (McPherson et al. 2017). Ensuring facilitators are equipped with the necessary skills and tools to adapt programs whilst simultaneously retaining program fidelity is essential for achieving both implementation and effectiveness outcomes (Mazzucchelli and Sanders 2010).

Third, a number of studies in this review discussed the lack of parent-child contact as a major barrier to program implementation. Difficulties associated with maintaining parent-child contact during imprisonment is common and is, arguably, one of the main reasons why parents and children have the potential to experience such profound difficulties when a parent is in prison (Poehlmann et al. 2008). Sporadic contact undermines the aims of parenting programs by limiting opportunities for parents to practice and develop the skills taught within the program. This may be particularly detrimental for programs that rely on play therapy such as PCIT and Filial Therapy (Scudder et al. 2014; Harris and Landreth 1997; Landreth and Lobaugh 1998). Future research should explore in more detail why establishing and/or maintaining contact is difficult to ensure that appropriate solutions can be implemented. For example, if prison regulations prevent regular contact it may be necessary to establish, what was referred to in some of the studies as, ‘enhanced visitation’ options for parents. If, on the other hand, barriers to contact exist at the level of the family (e.g., as distance from prison, family circumstances, relationship between caregivers, child’s care status, and/or a general reluctance from parents or children) different solutions may need to be implemented. If direct contact is not possible, program developers may wish to incorporate additional practical components to encourage parents to practice their skills in different ways. Examples from studies in this review include increased use of role play (Miller et al. 2013) or the use of video messages generated by imprisoned parents (Kennon et al. 2009).

Finally, there were a number of questions raised by the findings of this review which could not be investigated based on the information provided. A number of studies in this review indicated that both program facilitators and recipients expressed a desire for further support; yet it was not clear from the information provided what type of support would be most appropriate or whether the willingness to engage in further support is exclusive to those who remain in custody. Based on the studies included in this review, there was some evidence that more intensive programs produced greater effects and that providing additional support to parents on release from prison could enhance the benefits of programs. However, previous research has provided mixed findings as to how adjunctive support benefits parents (Sanders et al. 2007) and there is generally insufficient evidence about how program intensity impacts outcomes for high risk, socially disadvantaged parents (McPherson et al. 2017). Future research may wish to address this explicitly as identifying and adapting programs based on consumer needs may increase implementation and program success (Sanders and Kirby 2012).

### Strengths and Limitations

The findings from this review provide an important evidence base for future development, evaluation and implementation of parenting programs for parents in the CJS. However, it is not without limitation. The majority of studies identified for inclusion were rated as low or moderate quality, they generally had small sample sizes, and they often failed to provide adequate details about the sample, program, analysis, and/or findings. Of particular note was the lack of information about the type of crime, length of prison sentence and type of prison the participants were remanded in. Linked to this there was little information across studies about whether or not programs were mandatory or not and whether participation was incentivized. Moreover, caution is advised when interpreting the findings related to effectiveness as many of the studies failed to employ rigorous research designs or to report on group × time effects. In addition, heterogeneity in the programs, the outcome variables and the tools used to measure these, alongside inadequate reporting of program detail, made it difficult to draw firm conclusions. Including evidence about the feasibility, appropriateness, and meaningfulness of programs was a strength of this review and by employing robust definitions of the terms we were able to provide useful insights regarding the implementation of programs. To ensure that important evidence is not overlooked it is essential that future research is explicit in describing the range of data generated.

In Conclusion, the findings of this review suggest that parenting programs can be effective for improving parenting knowledge and attitudes of parents in prison; however, there is little evidence regarding the impact of programs on children’s social, emotional, or behavioral development. A number of methodological limitations across the studies were highlighted and this indicates that there is a need for further high-quality studies to be undertaken. There is also a need for further empirical work relating to the feasibility, appropriateness, and meaningfulness of programs as this might help optimize the implementation of programs. The current body of evidence is not yet sufficient to answer questions relating to the types of program most likely to be effective for use in prisons and wider criminal justice contexts. Moreover, a greater emphasis needs to be placed on understanding the conditions in which programs are most likely to succeed.
